# Supplementary material for: Nanoscale self-organization and metastable non-thermal metallicity in Mott insulators
Source: Nat Commun. 2022 Jun 28;13:3730. doi: 10.1038/s41467-022-31298-0 (PMC9240065; doi:10.1038/s41467-022-31298-0)
Supplement: Supplementary file 1 — Supplementary information [file 41467_2022_31298_MOESM1_ESM.pdf]

SUPPLEMENTARY INFORMATION

Nanoscale self-organisation in Mott insulators: a pathway to metastable metallicity

SUPPLEMENTARY NOTE 1. TIME-RESOLVED PHOTOEMITTED ELECTRON MICROSCOPY (PEEM)

A. Spatial overlap and fluence estimation

The spatial overlap is obtained by exploiting non-linear photoemission from surface impurities, which takes place when the laser beam is focused within the PEEM field of view. By rastering the sample position and monitoring the intensity of the impurity-induced non-linear photoemission, it is possible to reconstruct the laser profile, under the approximation that the impurity is much smaller than the laser spot (see Supplementary Figure **4**). The bi-dimensional Lorentzian fit evidences a slightly elliptical spot size (96 $\pm$ 2 μm $\times$ 110 $\pm$ 5 μm). The pump size and position was also monitored in real time by imaging the spot at the sample position on a CCD camera placed outside the PEEM chamber. The size estimated by the imaging is within the error bars associated to the value estimated by the rastering technique.

The incident laser power (up to $\sim$ 480 mW) was measured by a power meter before entering the vacuum chamber. The losses from the windows and the focusing lens inside the vacuum chamber have been taken into account to evaluate the pump power impinging on the sample. The maximal estimated incident pump fluence on the sample is 22 $\pm$ 4 mJ/cm^2.

B. Temporal coincidence

The non-linear photoemission intensity $I_{\rm NLP}$ from surface impurities is suppressed when both the near-infrared pump and X-ray fields are spatially and temporally coincident. Exploiting this non-linear effect, it is possible to reconstruct the cross-correlation between pump and probe pulses by measuring the intensity of the non-linear photoemission $I_{\rm NLP}$ (see solid grey line in Supplementary Figure **4**, panel b)). In the inset, it is possible to observe the real-space temporal evolution of $\mathrm{I_{NLP}}$ from the small surface defect before, during and after the coincidence with the X-ray single pulse. The delay for which the minimum of $\mathrm{I_{NLP}}$ is measured is set as the time zero of the time-resolved PEEM experiment.

C. Acquisition and analysis of the TR-PEEM images

Both static and non-equilibrium time-resolved PEEM experiments were performed at two energies (520 eV, 518 eV) and in the Linear Horizontal (LH) s-polarization configuration at different time delays with respect to the laser pump excitation. The temporal dynamics of the photo-induced phase transition has been followed within a temporal window spanning up to 7 ns. For each time delay, the final PEEM image was the result of the difference between the images taken at 520 eV (maximum domain contrast) and at 518 eV (minimum domain contrast) after an average of 4 sets of 40 images each (10 sec acquisition time per image). Each set of 40 images has been collected within a sequence of not-ordered different time delays, to rule out experimental fluctuations (drift of the sample, change in the X-ray intensity, etc...). In addition, for each sequence of positive delays we took a negative delay (image taken before time zero) reference image, which was used to compensate for possible background variations.

In order to plot the dynamics of monoclinic domains, we define the contrast between dark and light domains as

$$\delta I = |I^{dark} - I^{light}| \tag{1}$$

where I^{dark} and I^{light} are, respectively, the photoemitted intensities of the two different monoclinic domains reported in grey scale in Supplementary Figure **4**(c). Finally, the relative contrast variation (%) shown in Fig. 9 of the main text is the result of the renormalization with respect to the negative time delay PEEM image of each delays sequence and it is defined as

$$\frac{\delta I(t) - \delta I(t < t_0)}{\delta I_(t < t_0)} \cdot 100 \tag{2}$$

where $\delta I(t < t_0)$ is the contrast between dark and light domains at negative time delays. The error bars ($\pm$ 3 %) shown in Fig. 9d were calculated by considering the standard deviation of $\delta I(t < t_0)$ for all the negative delays acquired during the experiment.

SUPPLEMENTARY NOTE 2. SPACE GROUP SYMMETRIES IN V_2O_3

In this section we briefly recall the symmetry properties of the corundum phase, space group $R\bar{3}c$, no. 167, and monoclinic one, space group $I2/a$ no. 15.
With the conventions in Supplementary Figure **3**, the hexagonal lattice vectors transform under C_{3z} as

$$C_{3z}\big(\mathbf{a}_H\big) = \mathbf{b}_H\,,\quad C_{3z}\big(\mathbf{b}_H\big) = -\mathbf{a}_H - \mathbf{b}_H\,, \tag{3}$$

where we take, in accordance with the main text, $\mathbf{a}_H = a_H\,(1,0,0)$ and $\mathbf{b}_H = a_H\,(-1/2, \sqrt{3}/2, 0)$.

The V atoms are at the Wyckoff positions 12c, $(0,0,z)$, $(0,0,-z+1/2)$, $(0,0,-z)$ and $(0,0,z+1/2)$, with $z = 0.346$, plus the equivalent ones obtained summing $(0,0,0)$ or $(2/3,1/3,1/3)$ or $(1/3,2/3,2/3)$. We define $z = 1/3+\delta$, with $\delta = 0.0127$ the offset with respect to the basal plane, so that the 12c positions are, in ascending order along $\mathbf{c}_H$:

$$\begin{aligned}
c(1) &= (2/3,1/3,-\delta), & c(2) &= (1/3,2/3,\delta), & &\text{1st plane } A-\\
c(3) &= (0,0,1/6-\delta), & c(4) &= (2/3,1/3,1/6+\delta), & &\text{2nd plane } B+\\
c(5) &= (1/3,2/3,1/3-\delta), & c(6) &= (0,0,1/3+\delta), & &\text{3rd plane } C-\\
c(7) &= (2/3,1/3,1/2-\delta), & c(8) &= (1/3,2/3,1/2+\delta), & &\text{4th plane } A+\\
c(9) &= (0,0,2/3-\delta), & c(10) &= (1/3,2/3,\delta), & &\text{5th plane } B-\\
c(11) &= (1/3,2/3,5/6-\delta), & c(12) &= (0,0,5/6+\delta), & &\text{6th plane } C+\,.
\end{aligned} \tag{4}$$

The oxygen atoms instead occupy the 18e positions, $(x,0,1/4)$, $(0,x,1/4)$, $(-x,-x,1/4)$, $(-x,0,3/4)$, $(0,-x,3/4)$, and $(x,x,3/4)$, plus equivalent ones, with $x = 1/3$. It follows that on top of $c(3)$, at a distance $1/6+1/12 = 1/4$, there is a triangle of oxygens pointing in the $+\mathbf{x}$ direction, with the vertices at $x\,(1,0)$, $x\,(-1/2,\sqrt{3}/2)$ and $x\,(-1/2,-\sqrt{3}/2)$. The sign plus or minus in (4) indicate the directions along $\mathbf{x}$ in which the oxygen triangle on top of V points, while the letter is the stacking type.

The positions of V and O atoms on each layer are shown in Supplementary Figure **3**. There, we also shown the in-plane motion of V in the monoclinic phase, assuming $\mathbf{a}_H = \mathbf{b}_m$. Note that the motion is determined once the $\mathbf{b}_m$ axis is chosen: opposite V atoms surrounding the triangle void, see Supplementary Figure **3** move towards each

other, namely towards the centre of that triangular void.

The 120° rotation around z can be represented by the matrix

$$C_{3(001)} = \begin{pmatrix} 0 & -1 & 0 & 0 \\ 1 & -1 & 0 & 0 \\ 0 & 0 & 1 & 0 \end{pmatrix} , \tag{5}$$

where the first three columns describe how the transformation acts on the hexagonal lattice vectors, while the fourth is the translation that accompanies the symmetry operation. There are twofold axes, e.g., $(1, 1, 0)$, which interchanges $\mathbf{a}_H$ and $\mathbf{b}_H$. However, such symmetry operations must be accompanied by a translation of $1/2$ along $\mathbf{c}_H$, because of the oxygens arrangement, thus

$$C_{2(110)} = \begin{pmatrix} 0 & 1 & 0 & 0 \\ 1 & 0 & 0 & 0 \\ 0 & 0 & -1 & 1/2 \end{pmatrix} . \tag{6}$$

Similarly,

$$C_{2(100)} = \begin{pmatrix} 1 & -1 & 0 & 0 \\ 0 & -1 & 0 & 0 \\ 0 & 0 & -1 & 1/2 \end{pmatrix} . \tag{7}$$

Such rotations, which we denote as C_2', exchanges same type of planes, e.g., $A+$ with $A-$, and, within each plane, the sublattice.

In addition, there is a C_m symmetry corresponding to mirror planes perpendicular to the hexagonal axes. For instance the mirror plane perpendicular to $(1, 1, 0)$ has the following representation

$$C_{m(110)} = \begin{pmatrix} 0 & -1 & 0 & 0 \\ -1 & 0 & 0 & 0 \\ 0 & 0 & 1 & 1/2 \end{pmatrix} , \tag{8}$$

or that perpendicular to $(1, 0, 0)$, i.e., the $\mathbf{y} - \mathbf{z}$ plane,

$$C_{m(100)} = \begin{pmatrix} -1 & 1 & 0 & 0 \\ 0 & 1 & 0 & 0 \\ 0 & 0 & 1 & 1/2 \end{pmatrix} . \tag{9}$$

C_m symmetry operation exchanges same type of planes, e.g., $A+$ with $A-$, but it is diagonal in the sublattice. Note that $C_2' \otimes C_m$ gives inversion symmetry I, and, in particular, relates to each other different sublattices within each plane. We can therefore generate the whole space group using, besides the identity, C_{3z}, $C_2' = C_{2(110)}$, and the inversion I.

In addition there are two fractional translations $t(2/3, 1/3, 1/3)$ and $t(1/3, 2/3, 2/3)$ that relate to each other $A-$, $B-$ and $C-$, as well as $A+$, $B+$ and $C+$.

The monoclinic phase, space group $I2/a$ no. 15, has still inversion I. In addition it has a twofold axis corresponding to the monoclinic $\mathbf{b}_m$, accompanied by a 1/2 translation along $\mathbf{a}_m$. Specifically, if we assume $\mathbf{b}_m = \mathbf{a}_H$, then

$$\begin{aligned} \mathbf{a}_m &= \frac{2}{3}\,\mathbf{a}_H + \frac{4}{3}\,\mathbf{b}_H + \frac{1}{3}\,\mathbf{c}_H\,, \\ \mathbf{b}_m &= \mathbf{a}_H\,, \\ \mathbf{c}_m &= \frac{1}{3}\,\mathbf{a}_H + \frac{2}{3}\,\mathbf{b}_H - \frac{1}{3}\,\mathbf{c}_H\,. \end{aligned} \tag{10}$$

Therefore the translation corresponds to

$$\frac{1}{2}\,\mathbf{a}_m = \frac{1}{3}\,\mathbf{a}_H + \frac{2}{3}\,\mathbf{b}_H + \frac{1}{6}\,\mathbf{c}_H\,, \tag{11}$$

so that, in pseudo hexagonal representation,

$$C_{2b_m} = \begin{pmatrix} 1 & -1 & 0 & 1/3 \\ 0 & -1 & 0 & 2/3 \\ 0 & 0 & -1 & 1/6 \end{pmatrix}\,, \tag{12}$$

namely, because $1/2 + 2/3 = 7/6 \equiv 1/6$, it corresponds to $C_{2(100)}$ plus the translation $t(1/3, 2/3, 2/3)$. Since there is still the inversion

$$I = \begin{pmatrix} -1 & 0 & 0 & 0 \\ 0 & -1 & 0 & 0 \\ 0 & 0 & -1 & 0 \end{pmatrix}\,, \tag{13}$$

there exists a mirror plane perpendicular to $\mathbf{b}_m = \mathbf{a}_H$, which corresponds to the $\mathbf{a}_m - \mathbf{c}_m$ plane, whose representation is

$$C_{m(100)} = \begin{pmatrix} -1 & 1 & 0 & 1/3 \\ 0 & 1 & 0 & 2/3 \\ 0 & 0 & 1 & 1/6 \end{pmatrix}\,. \tag{14}$$

SUPPLEMENTARY NOTE 3. X-RAY LINEAR DICHROISM IN THE MONOCLINIC PHASE

In this section we study the origin and behaviour of the linear dichroic signal in the monoclinic phase of V_2O_3.

First, we find it worth deriving the explicit expression of the t_{2g} orbitals in the reference frame used in the main text. We consider a V atom surrounded by an octahedron of oxygens, see Supplementary Figure **1**. The V is at the origin, while, in units of the side of the octahedron, the basal plane oxygens are at positions $\mathrm{O}_1(1) = (0.5, 0.5, 0)$, $\mathrm{O}_2(1) = (-0.5, 0.5, 0)$, $\mathrm{O}_1(2) = (-0.5, -0.5, 0)$ and $\mathrm{O}_2(2) = (0.5, -0.5, 0)$, while the apical ones at $O'(1) = (0, 0, z)$ and $O'(2) = (0, 0, -z)$, where $z = 1/\sqrt{2}$. We denote the reference frame of the octahedron as (ξ, y, ζ), at the beginning coinciding with (x, y, z), in which the t_{2g} orbitals read

$$\phi_{\xi\zeta} = \xi\,\zeta\,, \quad \phi_{y\zeta} = y\,\zeta\,, \quad \phi_{\xi^2-y^2} = \frac{1}{2}\left(\xi^2 - y^2\right), \tag{15}$$

and the e_g ones as

$$\phi_{\xi y} = \xi\,y\,, \quad \phi_{\zeta^2} = \frac{1}{2\sqrt{3}}\left(2\zeta^2 - \xi^2 - y^2\right). \tag{16}$$

Next we rotate the octahedron by an angle ω around the y-axis, see Supplementary Figure **1**, which is the same as rotating the frame (ξ, y, ζ), namely

$$\boldsymbol{\xi} = \cos\omega\,\mathbf{x} - \sin\omega\,\mathbf{z}\,, \quad \mathbf{y} = \mathbf{y}\,, \quad \boldsymbol{\zeta} = \sin\omega\,\mathbf{x} + \cos\omega\,\mathbf{z}\,. \tag{17}$$

It follows that

$$\begin{aligned} O_2(1) &= -\frac{1}{2}\,\boldsymbol{\xi} + \frac{1}{2}\,\mathbf{y} \to -\frac{\cos\omega}{2}\,\mathbf{x} + \frac{1}{2}\,\mathbf{y} + \frac{\sin\omega}{2}\,\mathbf{z}\,,\\ O'(1) &= \frac{1}{\sqrt{2}}\,\boldsymbol{\zeta} \to \frac{\sin\omega}{\sqrt{2}}\,\mathbf{x} + \frac{\cos\omega}{\sqrt{2}}\,\mathbf{z}\,. \end{aligned} \tag{18}$$

We require the two oxygens to lie in a horizontal plane, which implies that

$$\cos\omega = \sqrt{\frac{1}{3}}\,, \quad \sin\omega = \sqrt{\frac{2}{3}}\,. \tag{19}$$

This actually corresponds to the top triangle of oxygens pointing in the $+\mathbf{x}$ direction. The opposite case of the triangle pointing in the $-\mathbf{x}$ direction corresponds to a rotation

by $-\omega$.

It follows that

$$\begin{aligned}
\phi_{y\zeta} &= \sin\omega\ \phi_{xy} + \cos\omega\ \phi_{yz}\ , \\
\phi_{\xi\zeta} &= -\frac{\sqrt{3}}{2}\ \sin 2\omega\ \phi_{z^2} + \frac{1}{2}\ \sin 2\omega\ \phi_{x^2-y^2} + \cos 2\omega\ \phi_{xz}\ , \\
\phi_{\xi^2-y^2} &= \frac{\sqrt{3}}{2}\ \sin^2\omega\ \phi_{z^2} + \frac{1+\cos^2\omega}{2}\ \phi_{x^2-y^2} - \frac{\sin 2\omega}{2}\ \phi_{xz}\ .
\end{aligned} \tag{20}$$

Because of the C_{3z} symmetry, is actually convenient to use linear combinations that transform like the irreps, one invariant under that symmetry, and the other two transforming as x and y. One readily finds that they actually are

$$\begin{aligned}
\phi_1 &= \sqrt{\frac{2}{3}}\ \phi_{xy} + \sqrt{\frac{1}{3}}\ \phi_{yz} \\
\phi_2 &= \sqrt{\frac{2}{3}}\ \phi_{x^2-y^2} - \sqrt{\frac{1}{3}}\ \phi_{xz}\ , \\
\phi_3 &= \phi_{z^2}\ ,
\end{aligned} \tag{21}$$

where $\phi_1 \sim x$, $\phi_2 \sim y$, and ϕ_3 invariant. For the triangle pointing in the $-\mathbf{x}$ direction,

$$\begin{aligned}
\phi_1' &= -\sqrt{\frac{2}{3}}\ \phi_{xy} + \sqrt{\frac{1}{3}}\ \phi_{yz} \\
\phi_2' &= \sqrt{\frac{2}{3}}\ \phi_{x^2-y^2} + \sqrt{\frac{1}{3}}\ \phi_{xz}\ , \\
\phi_3' &= \phi_{z^2}\ ,
\end{aligned} \tag{22}$$

which are related to the previous ones by $C_{2(100)}$, i.e., $y \to -y$ and $z \to -z$.

Now, we consider the L-edge x-ray absorption spectrum (XAS). The pre L_2-edge that we exploit in the main text corresponds to transferring a $2p$ electron in the $J = 1/2$ configuration into the t_{2g} shell, see Ref. [2]. The $J = 1/2$ $2p$ wavefunctions are

$$\begin{aligned}
\phi_{+1/2} &= -\sqrt{\frac{1}{3}}\ \phi_0\,\chi_\uparrow + \sqrt{\frac{2}{3}}\ \phi_{+1}\,\chi_\downarrow \\
&= -\sqrt{\frac{1}{3}}\ \phi_z\,\chi_\uparrow + \sqrt{\frac{1}{3}}\ \Big(-\phi_x\,\chi_\downarrow - i\,\phi_y\,\chi_\downarrow \Big)\ , \\
\phi_{-1/2} &= \sqrt{\frac{1}{3}}\ \phi_0\,\chi_\downarrow - \sqrt{\frac{2}{3}}\ \phi_{-1}\,\chi_\uparrow \\
&= \sqrt{\frac{1}{3}}\ \phi_z\chi_\downarrow - \sqrt{\frac{1}{3}}\ \Big(\phi_x\,\chi_\uparrow - i\,\phi_y\,\chi_\uparrow \Big)\ .
\end{aligned} \tag{23}$$

Apart from an overall constant, one readily finds that

$$
\begin{aligned}
x\,\phi_{+1/2} &= \tau\,\frac{1}{3}\,\phi_2\,\chi_\uparrow - \frac{\sqrt{2}}{3}\,\phi_2\,\chi_\downarrow + \sqrt{\frac{1}{3}}\,\phi_3\,\chi_\downarrow - i\,\tau\,\frac{\sqrt{2}}{3}\,\phi_1\,\chi_\downarrow\,,\\
y\,\phi_{+1/2} &= -\frac{1}{3}\,\phi_1\,\chi_\uparrow - \tau\,\frac{\sqrt{2}}{3}\,\phi_1\,\chi_\downarrow + i\,\frac{\sqrt{2}}{3}\,\phi_2\,\chi_\downarrow + i\,\sqrt{\frac{1}{3}}\,\phi_3\,\chi_\downarrow\,,\\
z\,\phi_{+1/2} &= -2\sqrt{\frac{1}{3}}\,\phi_3\,\chi_\uparrow + \tau\,\frac{1}{3}\,\phi_2\,\chi_\downarrow - i\,\frac{1}{3}\,\phi_1\,\chi_\downarrow\,,
\end{aligned}
\tag{24}
$$

as well as

$$
\begin{aligned}
x\,\phi_{-1/2} &= -\tau\,\frac{1}{3}\,\phi_2\,\chi_\downarrow - \frac{\sqrt{2}}{3}\,\phi_2\,\chi_\uparrow + \sqrt{\frac{1}{3}}\,\phi_3\,\chi_\uparrow + i\,\tau\,\frac{\sqrt{2}}{3}\,\phi_1\,\chi_\uparrow\,,\\
y\,\phi_{-1/2} &= \frac{1}{3}\,\phi_1\,\chi_\downarrow - \tau\,\frac{\sqrt{2}}{3}\,\phi_1\,\chi_\uparrow - i\,\frac{\sqrt{2}}{3}\,\phi_2\,\chi_\uparrow - i\,\sqrt{\frac{1}{3}}\,\phi_3\,\chi_\uparrow\,,\\
z\,\phi_{-1/2} &= 2\sqrt{\frac{1}{3}}\,\phi_3\,\chi_\downarrow + \tau\,\frac{1}{3}\,\phi_2\,\chi_\uparrow + i\,\frac{1}{3}\,\phi_1\,\chi_\uparrow\,,
\end{aligned}
\tag{25}
$$

where $\tau = +1$ for the top oxygen triangle pointing in the $+\mathbf{x}$ direction, and $\tau = -1$ otherwise.

The XAS corresponding to a given polarisation $\boldsymbol{\epsilon}$ is the expectation value of the operator

$$
A_{\boldsymbol{\epsilon}} \equiv Q_{\boldsymbol{\epsilon},+1/2}\,Q^\dagger_{\boldsymbol{\epsilon},+1/2} + Q_{\boldsymbol{\epsilon},-1/2}\,Q^\dagger_{\boldsymbol{\epsilon},-1/2}\,. \tag{26}
$$

For σ polarisation, i.e., $\boldsymbol{\epsilon} = \mathbf{z}$,

$$
\begin{aligned}
Q_{\mathbf{z},+1/2} &= -2\sqrt{\frac{1}{3}}\,d_{3\uparrow} + \tau\,\frac{1}{3}\,d_{2\downarrow} - i\,\frac{1}{3}\,d_{1\downarrow}\,,\\
Q_{\mathbf{z},-1/2} &= 2\sqrt{\frac{1}{3}}\,d_{3\downarrow} + \tau\,\frac{1}{3}\,d_{2\uparrow} + i\,\frac{1}{3}\,d_{1\uparrow}\,.
\end{aligned}
\tag{27}
$$

For π polarisation with $\boldsymbol{\epsilon} = \cos\phi\,\mathbf{x} + \sin\phi\,\mathbf{y}$,

$$
\begin{aligned}
Q_{\phi,+1/2} &= \frac{1}{3}\left[\,-\sin\phi\,d_{1\uparrow} + \tau\,\cos\phi\,d_{2\uparrow}\right]\\
&\quad + \frac{1}{3}\left[\,-i\,\tau\,\sqrt{2}\,\mathrm{e}^{-i\phi}\,d_{1\downarrow} - \sqrt{2}\,\mathrm{e}^{-i\phi}\,d_{2\downarrow} + \sqrt{3}\,\mathrm{e}^{i\phi}\,d_{3\downarrow}\right],\\
Q_{\phi,-1/2} &= \frac{1}{3}\left[\,\sin\phi\,d_{1\downarrow} - \tau\,\cos\phi\,d_{2\downarrow}\right]\\
&\quad + \frac{1}{3}\left[\,i\,\tau\,\sqrt{2}\,\mathrm{e}^{i\phi}\,d_{1\uparrow} - \sqrt{2}\,\mathrm{e}^{i\phi}\,d_{2\uparrow} + \sqrt{3}\,\mathrm{e}^{-i\phi}\,d_{3\uparrow}\right].
\end{aligned}
\tag{28}
$$

The monoclinic distortion generates a hybridisation between ϕ_2, actually its ϕ_{yz} component, which is the same for both oxygen surroundings, and ϕ_3. Such hybridisation is

proportional to the displacement along $\mathbf{y}$ times the offset with respect to the basal plane. Since V atoms above and below the basal plane move in opposite directions along $\mathbf{y}$, the hybridisation is the same for all of them. It follows that the local density matrix has diagonal elements only in the orbital index 1. This is true so long as we neglect spin orbit effects in the d-shell, which we shall do. Therefore, keeping only the operators that may have non-zero expectation values, and dropping a factor in front, we find

$$\begin{aligned} A^\pi_\phi &\equiv Q_{\phi,+1/2}\,Q^\dagger_{\phi,+1/2} + Q_{\phi,-1/2}\,Q^\dagger_{\phi,-1/2} \\ &= \Big(2+\sin^2\phi\Big)\sum_\sigma d_{1\sigma}\,d^\dagger_{1\sigma} + \Big(2+\cos^2\phi\Big)\sum_\sigma d_{2\sigma}\,d^\dagger_{2\sigma} + 3\sum_\sigma d_{3\sigma}\,d^\dagger_{3\sigma} \\ &\quad - \sqrt{6}\sum_\sigma \Big[\mathrm{e}^{2i\,\sigma\phi}\,d_{2\sigma}\,d^\dagger_{3\sigma} + \mathrm{e}^{-2i\,\sigma\phi}\,d_{3\sigma}\,d^\dagger_{2\sigma}\Big] \\ &\quad + \tau\,\cos\phi\,\sqrt{3}\sum_\sigma \sigma\Big[\mathrm{e}^{-i\,\sigma\phi}\,d_{2\sigma}\,d^\dagger_{3-\sigma} + \mathrm{e}^{i\,\sigma\phi}\,d_{3-\sigma}\,d^\dagger_{2\sigma}\Big]\,, \\ A^\sigma &\equiv Q_{\mathbf{z},+1/2}\,Q^\dagger_{\mathbf{z},+1/2} + Q_{\mathbf{z},-1/2}\,Q^\dagger_{\mathbf{z},-1/2} \\ &= \sum_\sigma d_{1\sigma}\,d^\dagger_{1\sigma} + \sum_\sigma d_{2\sigma}\,d^\dagger_{2\sigma} + 12\sum_\sigma d_{3\sigma}\,d^\dagger_{3\sigma} \\ &\qquad + 2\,\tau\,\sqrt{3}\sum_\sigma \sigma\Big(d_{2\sigma}\,d^\dagger_{3-\sigma} + d_{3-\sigma}\,d^\dagger_{2\sigma}\Big)\,. \end{aligned} \tag{29}$$

In the monoclinic phase, we can assume that one electron occupies orbital 1. Concerning orbitals 2 and 3, they are mixed together in the monoclinic phase,

$$d_{2\sigma} \to \cos\theta\,d_{b\sigma} - \sin\theta\,d_{a\sigma}\,,\quad d_{3\sigma} \to \sin\theta\,d_{b\sigma} + \cos\theta\,d_{a\sigma}\,, \tag{30}$$

with mixing angle $\theta \gtrsim 0$. We assume that the bonding combination, $d_{b\sigma}$, is occupied by one electron, while the anti bonding one, $d_{a\sigma}$, is empty. It follows that

$$\begin{aligned} &\sum_\sigma \langle\, d_{1\sigma}\,d^\dagger_{1\sigma}\,\rangle = 1\,, \\ &\sum_\sigma \langle\, d_{2\sigma}\,d^\dagger_{2\sigma}\,\rangle = \cos^2\theta + 2\sin^2\theta = 1+\sin^2\theta\,, \\ &\sum_\sigma \langle\, d_{3\sigma}\,d^\dagger_{3\sigma}\,\rangle = \sin^2\theta + 2\cos^2\theta = 2-\sin^2\theta\,, \\ &\sum_\sigma \langle\, d_{2\sigma}\,d^\dagger_{3\sigma}\,\rangle = \sum_\sigma \langle\, d_{3\sigma}\,d^\dagger_{2\sigma}\,\rangle = -\sin\theta\,\cos\theta\,. \end{aligned} \tag{31}$$

Neglecting spin orbit effects, the spin dependent terms cancel out in the antiferromagnetic configuration, so that the net result is

$$A^\pi_\phi = 11 - \sin^2\phi\,\sin^2\theta + \sqrt{6}\;\cos 2\phi\,\sin 2\theta\,,\quad A^\sigma = 26 - 11\,\sin^2\theta\,, \tag{32}$$

thus

$$\begin{aligned} A^\sigma - A^\pi_\phi &= 15 - 11\,\sin^2\theta + \sin^2\phi\,\sin^2\theta - \sqrt{6}\,\;\cos 2\phi\,\sin 2\theta \\ &\simeq 15 - 2\theta\,\sqrt{6}\,\;\cos 2\phi\,, \end{aligned} \tag{33}$$

the last equation valid at leading order in θ. Therefore, the x-ray linear dichroic signal is

$$\text{XLD} = A^\sigma - A^\pi_\phi \simeq 15 - 2\theta\,\sqrt{6}\,\;\cos 2\phi\,, \tag{34}$$

and is minimum for in-plane fields along the $\mathbf{b}_m$ axis, $\phi = 0, \pi$, and maximum for fields perpendicular to $\mathbf{b}_m$, $\phi = \pm\pi/2$, i.e., parallel to the $\mathbf{a}_m - \mathbf{c}_m$ monoclinic plane. We emphasise that the finite XLD signal that we have obtained is not due to magnetism, but only to the directionality of the t_{2g} orbitals. Magnetism can contribute to the signal, but we expect its contribution negligible because of the weak spin-orbit coupling in the $3d$-shell.

Now suppose that a pulsed laser pump transfers a percentage of e^π_g electrons into the a_{1g} orbital. Assuming that the e^π_g orbitals are equally depleted, thus

$$\sum_\sigma \langle\, d^\dagger_{1\sigma}\, d_{1\sigma}\,\rangle = 1 - \frac{\delta n}{2}\,,\quad \sum_\sigma \langle\, d^\dagger_{2\sigma}\, d_{2\sigma}\,\rangle = 1 - \frac{\delta n}{2}\,,\quad \sum_\sigma \langle\, d^\dagger_{3\sigma}\, d_{3\sigma}\,\rangle = \delta n\,,$$

with $\delta n > 0$, Supplementary Eq. (31) up to first order in θ changes into

$$\begin{aligned} &\sum_\sigma \langle\, d_{1\sigma}\, d^\dagger_{1\sigma}\,\rangle = 1 + \frac{\delta n}{2}\,,\quad \sum_\sigma \langle\, d_{2\sigma}\, d^\dagger_{2\sigma}\,\rangle \simeq 1 + \frac{\delta n}{2}\,,\\ &\sum_\sigma \langle\, d_{3\sigma}\, d^\dagger_{3\sigma}\,\rangle \simeq 2 - \delta n\,,\qquad \sum_\sigma \langle\, d_{2\sigma}\, d^\dagger_{3\sigma}\,\rangle \simeq -\theta\,\left(1 - \frac{3}{2}\,\delta n\right), \end{aligned} \tag{35}$$

and, accordingly,

$$\text{XLD} = A^\sigma - A^\pi_\phi \simeq 15 - 10\,\delta n + 2\theta\,\sqrt{6}\,\;\cos 2\phi\left(1 - \frac{3}{2}\,\delta n\right). \tag{36}$$

Therefore, the overall intensity of the XLD signal diminishes, as well as the maximum contrast.

SUPPLEMENTARY NOTE 4. LANDAU-GINZBURG THEORY OF THE STRAIN DRIVEN RHOMBOHEDRAL-MONOCLINIC TRANSFORMATION

In this section we show the detailed derivation of the Landau-Ginzburg energy functional for the shear strain used in the main text.

A. Elastic energy

The infinitesimal strain tensor $\hat{\epsilon}$ is defined through its components

$$\epsilon_{ij}(\mathbf{r}) = \frac{1}{2}\left(\frac{\partial u_i(\mathbf{r})}{\partial r_j} + \frac{\partial u_j(\mathbf{r})}{\partial r_i}\right), \tag{37}$$

where $i, j = 1, 2, 3$ indicate the coordinates x, y and z in the chosen reference frame, and $\mathbf{u}(\mathbf{r})$ is the displacement field. It is physically more convenient to define new combinations of the strain components through

$$\begin{aligned}
\epsilon(\mathbf{r}) &= \frac{\epsilon_{11}(\mathbf{r}) + \epsilon_{22}(\mathbf{r})}{2}, \qquad \epsilon_{33}(\mathbf{r}) = \epsilon_3(\mathbf{r}),\\
\boldsymbol{\epsilon}_1(\mathbf{r}) &= \begin{pmatrix} \dfrac{\epsilon_{11}(\mathbf{r}) - \epsilon_{22}(\mathbf{r})}{2} \\ \epsilon_{12}(\mathbf{r}) \end{pmatrix} = \begin{pmatrix} \varepsilon_{1,1}(\mathbf{r}) \\ \varepsilon_{1,2}(\mathbf{r}) \end{pmatrix} = \epsilon_1(\mathbf{r}) \begin{pmatrix} \cos\phi_1(\mathbf{r}) \\ \sin\phi_1(\mathbf{r}) \end{pmatrix},\\
\boldsymbol{\epsilon}_2(\mathbf{r}) &= \begin{pmatrix} \epsilon_{31}(\mathbf{r}) \\ \epsilon_{23}(\mathbf{r}) \end{pmatrix} = \begin{pmatrix} \varepsilon_{2,1}(\mathbf{r}) \\ \varepsilon_{2,2}(\mathbf{r}) \end{pmatrix} = \epsilon_2(\mathbf{r}) \begin{pmatrix} \cos\phi_2(\mathbf{r}) \\ \sin\phi_2(\mathbf{r}) \end{pmatrix},
\end{aligned} \tag{38}$$

with $\epsilon_i(\mathbf{r}) = \left|\boldsymbol{\epsilon}_i(\mathbf{r})\right| \geq 0$ and $\phi_i(\mathbf{r}) \geq 0 \mod (2\pi)$, $i = 1, 2$, which transform under a rotation around $\mathbf{z}$ by a generic angle ϕ according to:

$$\begin{aligned}
&\epsilon(\mathbf{r}) \to \epsilon(\mathbf{r}), \quad \epsilon_1(\mathbf{r}) \to \epsilon_1(\mathbf{r}), \quad \epsilon_2(\mathbf{r}) \to \epsilon_2(\mathbf{r}), \quad \epsilon_3(\mathbf{r}) \to \epsilon_3(\mathbf{r}),\\
&\phi_1(\mathbf{r}) \to \phi_1(\mathbf{r}) + 2\phi, \qquad \phi_2(\mathbf{r}) \to \phi_2(\mathbf{r}) + \phi.
\end{aligned} \tag{39}$$

The symmetry $R\bar{3}c$ of the corundum structure implies that the elastic energy must be invariant under C_{3z} rotations around the $\mathbf{z}$-axis, as well as, assuming, as in the main text, that the hexagonal $\mathbf{a}_H$ lattice vector is parallel to $\mathbf{x}$, under C_{2x} rotation around $\mathbf{x}$ and its C_{3z} equivalent axes. Specifically, with the strain defined in Supplementary Eq. (38), C_{3z} corresponds to

$$\begin{aligned}
&\epsilon(\mathbf{r}) \to \epsilon(\mathbf{r}), \quad \epsilon_1(\mathbf{r}) \to \epsilon_1(\mathbf{r}), \quad \epsilon_2(\mathbf{r}) \to \epsilon_2(\mathbf{r}), \quad \epsilon_3(\mathbf{r}) \to \epsilon_3(\mathbf{r}),\\
&\phi_1(\mathbf{r}) \to \phi_1(\mathbf{r}) + \frac{4\pi}{3}, \qquad \phi_2(\mathbf{r}) \to \phi_2(\mathbf{r}) + \frac{2\pi}{3},
\end{aligned} \tag{40}$$

while C_{2x} to

$$\begin{aligned}
&\epsilon(\mathbf{r}) \to \epsilon(\mathbf{r})\,, \quad \epsilon_1(\mathbf{r}) \to \epsilon_1(\mathbf{r})\,, \quad \epsilon_2(\mathbf{r}) \to \epsilon_2(\mathbf{r})\,, \quad \epsilon_3(\mathbf{r}) \to \epsilon_3(\mathbf{r})\,,\\
&\phi_1(\mathbf{r}) \to -\phi_1(\mathbf{r})\,, \qquad \phi_2(\mathbf{r}) \to \pi - \phi_2(\mathbf{r})\,.
\end{aligned} \tag{41}$$

The elastic energy invariant under these transformations reads [3]

$$\begin{aligned}
E_0 = \int d\mathbf{r} \,\bigg[&\Big(c_{11}+c_{12}\Big)\,\epsilon(\mathbf{r})^2 + \frac{c_{33}}{2}\,\epsilon_3(\mathbf{r})^2 + 2\,c_{13}\,\epsilon(\mathbf{r})\,\epsilon_3(\mathbf{r}) + 2\,c_{44}\,\epsilon_2(\mathbf{r})^2\\
&+ \big(c_{11}-c_{12}\big)\,\epsilon_1(\mathbf{r})^2 + 4\,c_{14}\,\epsilon_1(\mathbf{r})\,\epsilon_2(\mathbf{r})\,\sin\big(\phi_1(\mathbf{r})+\phi_2(\mathbf{r})\big)\bigg]\,.
\end{aligned} \tag{42}$$

Values of the elastic constants reported at around 300K are [4–6]: $c_{33} \simeq 34.2$, $c_{11} \simeq 27.1$, $c_{44} \simeq 8.5$, $c_{13} \simeq 15.1$, $c_{12} \simeq 8.4$ and $c_{14} \simeq -2$. We observe, through the transformation (39), that for $c_{14} = 0$, the elastic energy (42) is actually invariant under a generic $U(1)$ rotation around $\mathbf{z}$. Only the finite value of c_{14} makes the energy invariant only under C_3 rotations.

If the transition is only driven by $\boldsymbol{\epsilon}_2(\mathbf{r})$, we should assume that in the monoclinic phase the coefficient c_{44} becomes negative, or better that

$$2\,c_{44}\,\epsilon_2(\mathbf{r})^2 \;\to\; \tau\,\epsilon_2(\mathbf{r})^2 + \mu\,\epsilon_2(\mathbf{r})^4\,, \tag{43}$$

with τ positive in the corundum phase and negative in the monoclinic one, in which case it becomes important for the stability to include the anharmonic fourth order term with $\mu > 0$.

The next observation is that, if the monoclinic axis is $\mathbf{x}$, the finite dimer tilting represented by $\epsilon_{23}(\mathbf{r}) < 0$, induces the elongation of that same axis, namely $\epsilon_{11}(\mathbf{r}) > 0$. By symmetry, this effect should correspond to an addition anharmonic term in the elastic energy $\propto \epsilon_{23}(\mathbf{r})^2\,\epsilon_{11}(\mathbf{r})$ with negative coupling constant, plus all C_{3z} rotations, thus, using Supplementary Eq. (38),

$$\delta E = \int d\mathbf{r}\,\Big[-2g\,\epsilon(\mathbf{r})\,\epsilon_2(\mathbf{r})^2 + g\,\epsilon_1(\mathbf{r})\,\epsilon_2(\mathbf{r})^2\,\cos\big(\phi_1(\mathbf{r}) - 2\phi_2(\mathbf{r})\big)\Big]\,, \tag{44}$$

which is invariant under the transformations (40) and (41), and also account for $\epsilon(\mathbf{r}) > 0$ once $\epsilon_2(\mathbf{r}) \neq 0$. The values of $\phi_2(\mathbf{r})$ and $\phi_1(\mathbf{r})$ found in the monoclinic phase, i.e.,

$$\begin{aligned}
&\phi_2(\mathbf{r}) = \frac{\pi}{6} + \frac{2\pi}{3}\,n\,, \qquad n = 0,1,2\,,\\
&\phi_1(\mathbf{r}) = 2\phi_2(\mathbf{r}) + (2m+1)\,\pi \mod (2\pi)\,,
\end{aligned} \tag{45}$$

indeed minimise δE. On the contrary, they would maximise the other coupling term in Supplementary Eq. (42) should c_{14} remain negative as in the corundum phase at high temperature. This suggests that c_{14} must change sign in the monoclinic phase. There are actually evidences, in particular in the Chromium doped compounds [5], that c_{14} does change sign already approaching the transition from the corundum phase. Hereafter, we thus assume $c_{14} > 0$.

Therefore, a preliminary expression of the Landau-Ginzburg functional is

$$\begin{aligned}
E = &\int d\mathbf{r}\,\Big(c_{11}+c_{12}\Big)\,\epsilon(\mathbf{r})^2 + \int d\mathbf{r}\,\frac{c_{33}}{2}\,\epsilon_3(\mathbf{r})^2 + \int d\mathbf{r}\,\big(c_{11}-c_{12}\big)\,\epsilon_1(\mathbf{r})^2 \\
&+ \int d\mathbf{r}\,\Big[\,+\tau\,\epsilon_2(\mathbf{r})^2 + \mu\,\epsilon_2(\mathbf{r})^4\Big] \\
&+ \int d\mathbf{r}\,\Bigg[2\,c_{13}\,\epsilon(\mathbf{r})\,\epsilon_3(\mathbf{r}) + 4\,c_{14}\,\epsilon_1(\mathbf{r})\,\epsilon_2(\mathbf{r})\,\sin\big(\phi_1(\mathbf{r})+\phi_2(\mathbf{r})\big) \\
&\qquad - 2g\,\epsilon(\mathbf{r})\,\epsilon_2(\mathbf{r})^2 + g\,\epsilon_1(\mathbf{r})\,\epsilon_2(\mathbf{r})^2\,\cos\big(\phi_1(\mathbf{r})-2\phi_2(\mathbf{r})\big)\Bigg] \\
= &\,E\big[\epsilon\big] + E\big[\epsilon_3\big] + E\big[\boldsymbol{\epsilon}_1\big] + E\big[\boldsymbol{\epsilon}_2\big] + V\big[\epsilon,\epsilon_3\,\boldsymbol{\epsilon}_1,\boldsymbol{\epsilon}_2\big]\,.
\end{aligned} \tag{46}$$

We remark that the above energy functional is independent on the sample geometry.

B. Saint-Venant Compatibility equations

The compatibility constraints in three dimensions derive from the Saint-Venant equation

$$W_{ij\,kl} \equiv \partial_k\,\partial_l\,\epsilon_{ij} + \partial_i\,\partial_j\,\epsilon_{kl} - \partial_j\,\partial_k\,\epsilon_{il} - \partial_i\,\partial_l\,\epsilon_{jk} = 0\,. \tag{47}$$

We note that

$$\begin{aligned}
&W_{ij\,ij} \equiv 0\,, \\
&W_{ij\,kl} = W_{ji\,lk} = W_{lk\,ji} = W_{kl\,ij} = -W_{il\,kj}\,.
\end{aligned} \tag{48}$$

It follows that there are only 6 independent components, generated, for $i \neq j \neq k$, by

$$\begin{aligned}
&W_{ii\,jj} = -W_{ij\,ji} = \partial_j^2\,\epsilon_{ii} + \partial_i^2\,\epsilon_{jj} - 2\,\partial_i\,\partial_j\,\epsilon_{ij}\,, \\
&W_{ii\,jk} = W_{ii\,kj} = W_{kj\,ii} = -W_{ik\,ji} = \partial_j\,\partial_k\,\epsilon_{ii} + \partial_i^2\,\epsilon_{jk} - \partial_i\,\partial_j\,\epsilon_{ik} - \partial_i\,\partial_k\,\epsilon_{ij}\,,
\end{aligned} \tag{49}$$

which explicitly are $W_{11\,22}$, $W_{22\,33}$, $W_{33\,11}$, $W_{11\,23}$, $W_{22\,31}$ and $W_{33\,12}$, leading to

$$\begin{aligned}
\partial_2^2\,\epsilon_{11}(\mathbf{r}) + \partial_1^2\,\epsilon_{22}(\mathbf{r}) - 2\,\partial_1\partial_2\,\epsilon_{12}(\mathbf{r}) &= 0\,,\\
\partial_3^2\,\epsilon_{22}(\mathbf{r}) + \partial_2^2\,\epsilon_{33}(\mathbf{r}) - 2\,\partial_2\partial_3\,\epsilon_{23}(\mathbf{r}) &= 0\,,\\
\partial_1^2\,\epsilon_{33}(\mathbf{r}) + \partial_3^2\,\epsilon_{11}(\mathbf{r}) - 2\,\partial_3\partial_1\,\epsilon_{31}(\mathbf{r}) &= 0\,,\\
\partial_2\,\partial_3\,\epsilon_{11}(\mathbf{r}) + \partial_1^2\,\epsilon_{23}(\mathbf{r}) - \partial_3\partial_1\,\epsilon_{12}(\mathbf{r}) - \partial_1\,\partial_2\,\epsilon_{31}(\mathbf{r}) &= 0\,,\\
\partial_3\,\partial_1\,\epsilon_{22}(\mathbf{r}) + \partial_2^2\,\epsilon_{31}(\mathbf{r}) - \partial_2\partial_3\,\epsilon_{12}(\mathbf{r}) - \partial_1\,\partial_2\,\epsilon_{23}(\mathbf{r}) &= 0\,,\\
\partial_1\,\partial_2\,\epsilon_{33}(\mathbf{r}) + \partial_3^2\,\epsilon_{12}(\mathbf{r}) - \partial_3\partial_1\,\epsilon_{23}(\mathbf{r}) - \partial_2\,\partial_3\,\epsilon_{31}(\mathbf{r}) &= 0\,.
\end{aligned} \tag{50}$$

In reality the last 3 are automatically satisfied if the first 3 are.

If we assume a C-plane oriented film geometry, the strain is constant in z, so that the derivatives with respect to it vanish, and the compatibility conditions simplify into

$$\begin{aligned}
\partial_2^2\,\epsilon_{11}(\mathbf{r}) + \partial_1^2\,\epsilon_{22}(\mathbf{r}) - 2\,\partial_1\partial_2\,\epsilon_{12}(\mathbf{r}) &= 0\,,\\
\partial_2^2\,\epsilon_{33}(\mathbf{r}) = \partial_1^2\,\epsilon_{33}(\mathbf{r}) = \partial_1\,\partial_2\,\epsilon_{33}(\mathbf{r}) &= 0\,,\\
\partial_1^2\,\epsilon_{23}(\mathbf{r}) - \partial_1\,\partial_2\,\epsilon_{31}(\mathbf{r}) &= 0\,,\\
\partial_1\,\partial_2\,\epsilon_{23}(\mathbf{r}) - \partial_2^2\,\epsilon_{31}(\mathbf{r}) &= 0\,,
\end{aligned} \tag{51}$$

with $\mathbf{r}$ now being the planar coordinate, which, through Supplementary Eq. (38), can be written as

$$\begin{aligned}
\left(\partial_1^2 - \partial_2^2\right)\varepsilon_{1,1}(\mathbf{r}) + 2\,\partial_1\,\partial_2\,\varepsilon_{1,2}(\mathbf{r}) &= \nabla^2\,\epsilon(\mathbf{r})\,,\\
\partial_2^2\,\epsilon_{33}(\mathbf{r}) = \partial_1^2\,\epsilon_{33}(\mathbf{r}) = \partial_1\,\partial_2\,\epsilon_{33}(\mathbf{r}) &= 0\,,\\
\partial_1\left(\partial_1\,\varepsilon_{2,2}(\mathbf{r}) - \partial_2\,\varepsilon_{2,1}(\mathbf{r})\right) = \partial_1\,\boldsymbol{\nabla}\wedge\boldsymbol{\epsilon}_2(\mathbf{r}) &= 0\,,\\
\partial_2\left(\partial_1\,\varepsilon_{2,2}(\mathbf{r}) - \partial_2\,\varepsilon_{2,1}(\mathbf{r})\right) = \partial_2\,\boldsymbol{\nabla}\wedge\boldsymbol{\epsilon}_2(\mathbf{r}) &= 0\,.
\end{aligned} \tag{52}$$

They are satisfied by a constant $\epsilon_{33}(\mathbf{r}) = \epsilon_{33}$, $\forall\,\mathbf{r}$, a curl free $\boldsymbol{\epsilon}_2(\mathbf{r})$, i.e.,

$$\boldsymbol{\nabla}\wedge\boldsymbol{\epsilon}_2(\mathbf{r}) = 0\,, \tag{53}$$

and, finally, by the constraint

$$\left(\partial_1^2 - \partial_2^2\right)\varepsilon_{1,1}(\mathbf{r}) + 2\,\partial_1\,\partial_2\,\varepsilon_{1,2}(\mathbf{r}) = \nabla^2\,\epsilon(\mathbf{r})\,, \tag{54}$$

which can be enforced adding a Lagrange multiplier and the following term to the elastic energy, written for a generic non-uniform strain

$$\begin{aligned}\delta E_{\text{compatibility}} &= \iint d\mathbf{r}\, d\mathbf{r}'\, \lambda(\mathbf{r}) \Big[R_1(\mathbf{r}-\mathbf{r}')\, \varepsilon_{1,1}(\mathbf{r}') + R_2(\mathbf{r}-\mathbf{r}')\, \varepsilon_{1,2}(\mathbf{r}') - \delta\big(\mathbf{r}-\mathbf{r}'\big)\, \epsilon(\mathbf{r}') \Big] \\ &= \frac{1}{V} \sum_{\mathbf{k}} \lambda(-\mathbf{k}) \Bigg[\frac{k_1^2 - k_2^2}{k_1^2 + k_2^2}\, \varepsilon_{1,1}(\mathbf{k}) + \frac{2\, k_1\, k_2}{k_1^2 + k_2^2}\, \varepsilon_{1,2}(\mathbf{k}) - \epsilon(\mathbf{k}) \Bigg] .\end{aligned} \tag{55}$$

Specifically, for a two dimensional coordinate $\mathbf{r} = r\,\big(\cos\phi, \sin\phi\big)$, and recalling the following equalities involving the Bessel functions

$$\begin{aligned} &\mathrm{e}^{ix\cos\theta} = J_0(x) + 2 \sum_{n\geq 1} i^n\, J_n(x)\, \cos n\theta\,, && \int_0^\infty dx\, J_n(x) = 1\,, \\ &2n\, J_n(x) = x\, J_{n-1}(x) + x\, J_{n+1}(x)\,, && x\, J_0(x) = \frac{d}{dx}\Big[x\, J_1(x)\Big]\,, \end{aligned} \tag{56}$$

we find

$$\begin{aligned} R_1(\mathbf{r}) &= \int_0^\infty \frac{k\, dk}{4\pi^2} \int_0^{2\pi} d\theta\, \mathrm{e}^{ikr\,\cos(\theta-\phi)}\, \cos 2\theta = \int_0^\infty \frac{k\, dk}{4\pi^2} \int_0^{2\pi} d\theta\, \mathrm{e}^{ikr\,\cos\theta}\, \cos 2\big(\theta+\phi\big) \\ &= \int_0^\infty \frac{k\, dk}{4\pi^2} \int_0^{2\pi} d\theta\, \mathrm{e}^{ikr\,\cos\theta}\, \cos 2\theta\, \cos 2\phi \\ &= -2\cos 2\phi \int_0^\infty \frac{k\, dk}{4\pi^2}\, J_2(kr) \int_0^{2\pi} d\theta\, \cos^2 2\theta = -\frac{\cos 2\phi}{2\pi\, r^2} \int_0^\infty dx\, x\, J_2(x) \\ &= -\frac{\cos 2\phi}{2\pi\, r^2} \int_0^\infty dx\, \Big(2J_1(x) - x\, J_0(x)\Big) = -\frac{\cos 2\phi}{\pi\, r^2}\,, \\ R_2(\mathbf{r}) &= \int_0^\infty \frac{k\, dk}{4\pi^2} \int_0^{2\pi} d\theta\, \mathrm{e}^{ikr\,\cos(\theta-\phi)}\, \sin 2\theta = \int_0^\infty \frac{k\, dk}{4\pi^2} \int_0^{2\pi} d\theta\, \mathrm{e}^{ikr\,\cos\theta}\, \sin 2\big(\theta+\phi\big) \\ &= -\frac{\sin 2\phi}{\pi\, r^2}\,, \end{aligned} \tag{57}$$

Equation (53) implies instead that, if $\boldsymbol{\epsilon}_2(\mathbf{r})$ changes abruptly across an interface, its component tangential to it must be continuous, which leads to the allowed interfaces in Supplementary Figure **2**.

C. Landau-Ginzburg functional

Since $\epsilon_{33} = \epsilon_3$ is constant in $\mathbf{r}$, we shall discard it in the following analysis. We then add to the elastic energy Supplementary Eq. (46) the term (55) that enforces the compatibility equations, and integrate out all strain components but the order parameter $\boldsymbol{\epsilon}_2(\mathbf{r})$.

The energy functionals of $\epsilon(\mathbf{r})$ and $\boldsymbol{\epsilon}_1(\mathbf{r})$ can be written as

$$E\big[\epsilon\big] = \int d\mathbf{r} \Bigg\{ \big(c_{11}+c_{12}\big)\,\epsilon(\mathbf{r})^2 - \Lambda(\mathbf{r})\,\epsilon(\mathbf{r}) \Bigg\},$$
$$E\Big[\boldsymbol{\epsilon}_1\Big] = \int d\mathbf{r} \Bigg\{ \big(c_{11}-c_{12}\big)\,\boldsymbol{\epsilon}_1(\mathbf{r})\cdot\boldsymbol{\epsilon}_1(\mathbf{r}) + \Big(\Lambda_1(\mathbf{r})\,\varepsilon_{1,1}(\mathbf{r}) + \Lambda_2(\mathbf{r})\,\varepsilon_{1,2}(\mathbf{r})\Big) \Bigg\}, \tag{58}$$

where

$$\Lambda(\mathbf{r}) = \lambda(\mathbf{r}) + 2g\,\epsilon_2(\mathbf{r})^2\,,$$
$$\Lambda_1(\mathbf{r}) = \int d\mathbf{r}'\,\lambda(\mathbf{r}')\,R_1(\mathbf{r}'-\mathbf{r}) + 4c_{14}\,\varepsilon_{2,2}(\mathbf{r}) + g\,\Big(\varepsilon_{2,1}(\mathbf{r})^2 - \varepsilon_{2,2}(\mathbf{r})^2\Big)\,, \tag{59}$$
$$\Lambda_2(\mathbf{r}) = \int d\mathbf{r}'\,\lambda(\mathbf{r}')\,R_2(\mathbf{r}'-\mathbf{r}) + 4c_{14}\,\varepsilon_{2,1}(\mathbf{r}) + 2g\,\varepsilon_{2,1}(\mathbf{r})\,\varepsilon_{2,2}(\mathbf{r})\,,$$

Integrating out ϵ and $\boldsymbol{\epsilon}_1$ leads to

$$\delta_\epsilon E\Big[\boldsymbol{\epsilon}_2,\lambda\Big] = -\frac{1}{4\,\big(c_{11}+c_{12}\big)}\int d\mathbf{r}\,\Lambda(\mathbf{r})^2\,,$$
$$\delta_{\boldsymbol{\epsilon}_1} E\Big[\boldsymbol{\epsilon}_2,\lambda\Big] = -\frac{1}{4\,\big(c_{11}-c_{12}\big)}\int d\mathbf{r}\,\Big[\,\Lambda_1(\mathbf{r})^2 + \Lambda_2(\mathbf{r})^2\,\Big]\,, \tag{60}$$

where, explicitly,

$$\Lambda(\mathbf{r})^2 = \lambda(\mathbf{r})^2 + 4g^2\,\epsilon_2(\mathbf{r})^4 + 4g\,\lambda(\mathbf{r})\,\epsilon_2(\mathbf{r})^2\,,$$
$$\Lambda_1(\mathbf{r})^2 + \Lambda_2(\mathbf{r})^2 = \lambda(\mathbf{r})^2 + 16\,c_{14}\,\boldsymbol{\epsilon}_2(\mathbf{r})\cdot\boldsymbol{\epsilon}_2(\mathbf{r}) + g^2\,\epsilon_2(\mathbf{r})^4 + 8\,c_{14}\,g\,\epsilon_2(\mathbf{r})^3\,\sin 3\phi_2(\mathbf{r})$$
$$+ \int d\mathbf{r}'\,\lambda(\mathbf{r}')\Bigg\{ R_2(\mathbf{r}'-\mathbf{r})\,\Big[8c_{14}\,\varepsilon_{2,1}(\mathbf{r}) + 4g\,\varepsilon_{2,1}(\mathbf{r})\,\varepsilon_{2,2}(\mathbf{r})\Big] \tag{61}$$
$$+ R_1(\mathbf{r}'-\mathbf{r})\,\Big[8c_{14}\,\varepsilon_{2,2}(\mathbf{r}) + 2g\,\Big(\varepsilon_{2,1}(\mathbf{r})^2 - \varepsilon_{2,2}(\mathbf{r})^2\Big)\Big]\Bigg\}.$$

The Lagrange multiplier is determined through the saddle point of the functional

$$F\big[\lambda\big] = \int d\mathbf{r}\Bigg\{ -\frac{1}{2}\,\frac{c_{11}}{c_{11}^2 - c_{12}^2}\,\lambda(\mathbf{r})^2 - \Omega(\mathbf{r})\,\lambda(\mathbf{r}) \Bigg\}, \tag{62}$$

where, by definition,

$$\Omega(\mathbf{r}) = \frac{g}{c_{11}+c_{12}}\,\epsilon_2(\mathbf{r})^2$$
$$+ \frac{1}{4\big(c_{11}-c_{12}\big)}\int d\mathbf{r}'\Bigg\{ R_2(\mathbf{r}-\mathbf{r}')\,\Big[8c_{14}\,\varepsilon_{2,1}(\mathbf{r}') + 4g\,\varepsilon_{2,1}(\mathbf{r}')\,\varepsilon_{2,2}(\mathbf{r}')\Big] \tag{63}$$
$$+ R_1(\mathbf{r}-\mathbf{r}')\,\Big[8c_{14}\,\varepsilon_{2,2}(\mathbf{r}') + 2g\,\Big(\varepsilon_{2,1}(\mathbf{r}')^2 - \varepsilon_{2,2}(\mathbf{r}')^2\Big)\Big]\Bigg\}$$
$$\equiv \frac{g}{c_{11}+c_{12}}\,\epsilon_2(\mathbf{r})^2 + \Omega_R(\mathbf{r})\,,$$

yielding the following contribution to the energy functional of $\boldsymbol{\epsilon}_2$

$$\begin{aligned}\delta E_\lambda\big[\boldsymbol{\epsilon}_2\big] &= \frac{c_{11}^2-c_{12}^2}{2c_{11}}\int d\mathbf{r}\,\Omega(\mathbf{r})^2 = \frac{g^2\big(c_{11}-c_{12}\big)}{2c_{11}\big(c_{11}+c_{12}\big)}\int d\mathbf{r}\,\epsilon_2(\mathbf{r})^4\\ &+\frac{g\big(c_{11}-c_{12}\big)}{c_{11}}\int d\mathbf{r}\,\epsilon_2(\mathbf{r})^2\,\Omega_R(\mathbf{r}) + \frac{c_{11}^2-c_{12}^2}{2c_{11}}\int d\mathbf{r}\,\Omega_R(\mathbf{r})^2\,.\end{aligned}\tag{64}$$

The last term favours the growth of domains [7]. In its presence, the amplitude of the strain $\epsilon_2(\mathbf{r}) = \big|\boldsymbol{\epsilon}_2(\mathbf{r})\big|$ would be constant everywhere but at the interfaces between domains, where it would vanish. Therefore, we can safely neglect the term $\propto \int \epsilon_2(\mathbf{r})^2\,\Omega_R(\mathbf{r})$, since either $\epsilon_2(\mathbf{r})$ is zero, or, when it is constant, it is the angular average of the kernels $R_1(\mathbf{r})$ and $R_2(\mathbf{r})$ that makes the integral to vanish.

Collecting all terms, and further adding a stiffness , we finally obtain the Landau-Ginzburg functional of the order parameter

$$\begin{aligned}E\Big[\boldsymbol{\epsilon}_2\Big] = \int d\mathbf{r}\Bigg\{ &-\frac{K}{2}\,\boldsymbol{\epsilon}_2(\mathbf{r})\cdot\nabla^2\boldsymbol{\epsilon}_2(\mathbf{r}) + \tau\,\boldsymbol{\epsilon}_2(\mathbf{r})\cdot\boldsymbol{\epsilon}_2(\mathbf{r}) + \frac{c_{11}^2-c_{12}^2}{2c_{11}}\,\Omega_R(\mathbf{r})^2\\ &-\gamma\,\epsilon_2(\mathbf{r})^3\,\sin 3\phi_2(\mathbf{r}) + \mu\,\Big(\boldsymbol{\epsilon}_2(\mathbf{r})\cdot\boldsymbol{\epsilon}_2(\mathbf{r})\Big)^2\Bigg\}\,,\end{aligned}\tag{65}$$

where $\mu > 0$ and $\gamma \propto c_{14}\,g$. The quadratic coupling constant τ is positive above the transition, and negative below. We mentioned that $\gamma > 0$ below the transition, but negative, and small in absolute value, quite above it, possibly vanishing at or right before the transition.

Let us now analyse in detail the term $\propto \int \Omega_R(\mathbf{r})^2$. Through (63), $\Omega_R(\mathbf{r})$ involves the two combinations

$$\begin{aligned}\Phi_1(\mathbf{r}) &= 8c_{14}\,\varepsilon_{2,1}(\mathbf{r}) + 4g\,\varepsilon_{2,1}(\mathbf{r})\,\varepsilon_{2,2}(\mathbf{r}) = 8c_{14}\,\epsilon_2(\mathbf{r})\,\cos\phi_2(\mathbf{r}) + 2g\,\epsilon_2(\mathbf{r})^2\,\sin 2\phi_2(\mathbf{r})\,,\\ \Phi_2(\mathbf{r}) &= 8c_{14}\,\varepsilon_{2,2}(\mathbf{r}) + 2g\Big(\varepsilon_{2,1}(\mathbf{r})^2 - \varepsilon_{2,2}(\mathbf{r})^2\Big) = 8c_{14}\,\epsilon_2(\mathbf{r})\,\sin\phi_2(\mathbf{r}) + 2g\,\epsilon_2(\mathbf{r})^2\,\cos 2\phi_2(\mathbf{r})\,.\end{aligned}\tag{66}$$

At the values of the angle ϕ_2 that minimise the energy, i.e., $\phi_2 = \pi/6,\, 5\pi/6,\, 3\pi/2$, $\Phi_1(\mathbf{r}) = \Phi(\mathbf{r})\,\cos\phi_2$ and $\Phi_2(\mathbf{r}) = \Phi(\mathbf{r})\,\sin\phi_2$. Therefore, without loss of generality, we can approximate $\Phi_1(\mathbf{r})$ and $\Phi_2(\mathbf{r})$ with $\varepsilon_{2,1}(\mathbf{r})$ and $\varepsilon_{2,2}(\mathbf{r})$, respectively, apart from

a proportionality constant that we will absorb into an effective coupling constant, so that

$$\frac{c_{11}^2 - c_{12}^2}{2c_{11}} \int d\mathbf{r}\, \Omega_R(\mathbf{r})^2 \simeq \kappa \iint d\mathbf{r}\, d\mathbf{r}'\, \boldsymbol{\epsilon}_2(\mathbf{r}) \cdot \begin{pmatrix} K_{11}(\mathbf{r}-\mathbf{r}') & K_{12}(\mathbf{r}-\mathbf{r}') \\ K_{21}(\mathbf{r}-\mathbf{r}') & K_{22}(\mathbf{r}-\mathbf{r}') \end{pmatrix} \boldsymbol{\epsilon}_2(\mathbf{r}')\,, \tag{67}$$

with $\kappa > 0$, and

$$\begin{aligned} K_{11}(\mathbf{r}) &= \frac{1}{V} \sum_{\mathbf{k}} \mathrm{e}^{i\mathbf{k}\cdot\mathbf{r}} \left(\frac{2k_1\, k_2}{k_1^2 + k_2^2} \right)^2 = -\cos 4\phi\, \frac{1}{\pi\, r^2}\,, \\ K_{22}(\mathbf{r}) &= \frac{1}{V} \sum_{\mathbf{k}} \mathrm{e}^{i\mathbf{k}\cdot\mathbf{r}} \left(\frac{k_1^2 - k_2^2}{k_1^2 + k_2^2} \right)^2 = \cos 4\phi\, \frac{1}{\pi\, r^2} = -K_{11}(\mathbf{r})\,, \\ K_{12}(\mathbf{r}) = K_{21}(\mathbf{r}) &= \frac{1}{V} \sum_{\mathbf{k}} \mathrm{e}^{i\mathbf{k}\cdot\mathbf{r}} \left(\frac{2k_1\, k_2}{k_1^2 + k_2^2} \right) \left(\frac{k_1^2 - k_2^2}{k_1^2 + k_2^2} \right) = \sin 4\phi\, \frac{1}{\pi\, r^2}\,. \end{aligned} \tag{68}$$

In conclusion, the Ginzburg-Landau functional reads

$$\begin{aligned} E\Big[\boldsymbol{\epsilon}_2\Big] \to \int d\mathbf{r} \Bigg\{ &- \frac{K}{2}\, \boldsymbol{\epsilon}_2(\mathbf{r}) \cdot \nabla^2 \boldsymbol{\epsilon}_2(\mathbf{r}) + \tau\, \boldsymbol{\epsilon}_2(\mathbf{r}) \cdot \boldsymbol{\epsilon}_2(\mathbf{r}) \\ &- \gamma\, \epsilon_2(\mathbf{r})^3 \sin 3\phi_2(\mathbf{r}) + \mu \Big(\boldsymbol{\epsilon}_2(\mathbf{r}) \cdot \boldsymbol{\epsilon}_2(\mathbf{r}) \Big)^2 \Bigg\} \\ &+ \kappa \iint d\mathbf{r}\, d\mathbf{r}'\, \boldsymbol{\epsilon}_2(\mathbf{r}) \cdot \begin{pmatrix} K_{11}(\mathbf{r}-\mathbf{r}') & K_{12}(\mathbf{r}-\mathbf{r}') \\ K_{21}(\mathbf{r}-\mathbf{r}') & K_{22}(\mathbf{r}-\mathbf{r}') \end{pmatrix} \boldsymbol{\epsilon}_2(\mathbf{r}')\,, \end{aligned} \tag{69}$$

with positive μ, γ and κ, supplemented by the condition Eq. (53). Supplementary Eq. (69) can describe a transition once τ changes sign from positive to negative. Since γ is likely very small near the structural transition, the latter might well be continuous or, if discontinuous, not as sharp as the jump in resistivity, as suggested by Ref. [8].

D. Mean-field approximation in the pseudo-spin representation

As discussed in the main text, the dimer stretching, main responsible of the metal-insulator transition, add a further term to the energy functional (69) that depends on a dimensionless field $\eta(\mathbf{r})$ and reads

$$\delta E\big[\epsilon_2, \eta\big] = a \int d\mathbf{r} \left[\left(\eta(\mathbf{r})^2 - \frac{1}{4} \right)^2 - g\Big(\epsilon_2(\mathbf{r})^2 - \epsilon_{MIT}^2 \Big)\, \eta(\mathbf{r}) \right], \tag{70}$$

with $a > 0$, $g > 0$, and ϵ^2_{MIT} either positive or negative. A minimum at $\eta < 0$ corresponds to a metal, while one at $\eta > 0$ to an insulator, and both may coexist. Note that $\epsilon_2^2 > \epsilon^2_{MIT}$ implies that the global minimum is insulating, whereas $\epsilon_2^2 < \epsilon^2_{MIT}$ that is metallic. Therefore, the total energy functional is the sum of the shear strain energy $E\big[\boldsymbol{\epsilon}_2\big]$ in Supplementary Eq. (69) and of the dimer stretching contribution $\delta E\big[\epsilon_2, \eta\big]$ in Supplementary Eq. (70), and is

$$E\big[\boldsymbol{\epsilon}_2, \eta\big] = E\big[\boldsymbol{\epsilon}_2\big] + \delta E\big[\epsilon_2, \eta\big]\,, \tag{71}$$

which can actually describe rhombohedral and monoclinic phases, either metallic or insulating, as those observed experimentally in and out-of equilibrium.

In order to study the free energy corresponding to the classical energy functional (71), we decided to use the mean-field theory developed in Refs. [9, 10].

For that, we consider a square film, and introduce a dense mesh of N sites to discretise the real space coordinate $\mathbf{r}$. Below the monoclinic insulator spinodal point, the landscape of the energy functional (71) displays more than a single potential well, corresponding, within the coexistence region, to the rhombohedral metal and the three equivalent monoclinic twins, and only to the latter three below the rhombohedral metal spinodal point. We expect that at thermal equilibrium the field variables, $\boldsymbol{\epsilon}_2(\mathbf{r})$ and $\eta(\mathbf{r})$, will locally fluctuate inside one of those potential wells. We henceforth neglect such thermal fluctuations, and constraint $\boldsymbol{\epsilon}_2(\mathbf{r})$ and $\eta(\mathbf{r})$ to locally take only the values at the minima of the potential wells. This is equivalent to assuming that the entropy is just contributed by the configurational term due to the presence of different domains.

Below the monoclinic insulator spinodal point we thus assume that

- the monoclinic insulating domains are characterised by

$$\boldsymbol{\epsilon}_2(\mathbf{r}) = \overline{\epsilon}_2(\tau)\, \mathbf{S}_n\,, \quad n = 1, 2, 3\,, \tag{72}$$

  where $\overline{\epsilon}_2(\tau)$ is the value of ϵ_2 at the monoclinic homogeneous minimum of Supplementary Eq. (71), even if metastable, with energy $E_m(\tau)$, and

$$\mathbf{S}_n = \begin{pmatrix} \cos\phi_{2,n} \\ \sin\phi_{2,n} \end{pmatrix}\,, \tag{73}$$

  are unit-length two-component vectors, with $\phi_{2,n}$, $n = 1, 2, 3$, the angles defined in Supplementary Eq. (45);

- the rhombohedral metallic domains are instead identified by

$$\boldsymbol{\epsilon}_2(\mathbf{r}) = \mathbf{0} = \overline{\epsilon}_2(\tau)\begin{pmatrix}0\\0\end{pmatrix} \equiv \overline{\epsilon}_2(\tau)\,\mathbf{S}_0\,, \tag{74}$$

with the same amplitude $\overline{\epsilon}_2(\tau)$ as in Supplementary Eq. (72), thus absorbing the vanishing strain into a zero-length spin. Its energy, E_r, is actually independent of the reduced temperature τ. We allow for rhombohedral domains also below the rhombohedral spinodal point because $\epsilon_2 = 0$ is always a turning point of Supplementary Eq. (71) [9, 10]. Therefore, it could be stabilised at the interface between two monoclinic domains, despite being a maximum of the energy.

In this approximation, the energy functional (71) below the monoclinic insulator spinodal point can be written as a pseudo-spin Hamiltonian

$$\begin{aligned} H(\tau) = &\int d\mathbf{r}\,\Big\{E(\tau)\,\mathbf{S}(\mathbf{r})\cdot\mathbf{S}(\mathbf{r}) - \overline{\epsilon}_2(\tau)^2\,\frac{K}{2}\,\mathbf{S}(\mathbf{r})\cdot\nabla^2\mathbf{S}(\mathbf{r})\Big\}\\ &+\kappa\,\overline{\epsilon}_2(\tau)^2\iint d\mathbf{r}\,d\mathbf{r}'\,\mathbf{S}(\mathbf{r})\cdot\,\hat{U}(\mathbf{r}-\mathbf{r}')\mathbf{S}(\mathbf{r}) \equiv \frac{1}{N}\sum_{\mathbf{q}}\mathbf{S}(\mathbf{q})\cdot\,\hat{H}(-\mathbf{q},\tau)\,\mathbf{S}(-\mathbf{q})\,, \end{aligned} \tag{75}$$

where $E(\tau) = E_m(\tau) - E_r$, and, we remind, $\mathbf{S}(\mathbf{r})$ can take only the values $\mathbf{S}_n$, $n = 0, \ldots, 3$, see Eqs. (73) and (74). We also note that the Hamiltonian (75) depends explicitly on temperature through the parameter τ. Following Refs. [9, 10] we treat such pseudo-spin Hamiltonian in mean field. Since the key role in the domain formation is played by the long range potential, which is diagonal in momentum space, we assume a factorised density matrix $\hat{\rho} = \prod_{\mathbf{q}}\hat{\rho}(\mathbf{q})$ that minimises the free-energy functional

$$F\big[\hat{\rho}\big] = \mathrm{Tr}\Big(\hat{\rho}\,H(\tau)\Big) - T\sum_{\mathbf{q}}\mathrm{Tr}\Big(\hat{\rho}(\mathbf{q})\,\ln\hat{\rho}(\mathbf{q})\Big)\,, \tag{76}$$

thus providing an upper estimate of the actual free energy. One readily finds that the minimum is obtained for

$$\begin{aligned} \hat{\rho} &= \exp\Big[-\frac{\beta}{N}\sum_{\mathbf{q}}\mathbf{S}(\mathbf{q})\cdot\mathbf{B}(-\mathbf{q},\tau)\Big]\\ &= \exp\Big[-\beta\int d\mathbf{r}\,\mathbf{S}(\mathbf{r})\cdot\mathbf{B}(\mathbf{r},\tau)\Big]\,, \end{aligned} \tag{77}$$

where

$$\mathbf{B}(\mathbf{r},\tau) = \frac{1}{N}\sum_{\mathbf{q}} e^{i\mathbf{q}\cdot\mathbf{r}}\,\hat{H}(\mathbf{q},\tau)\,\mathrm{Tr}\Big(\hat{\rho}(\mathbf{q})\,\mathbf{S}(\mathbf{q})\Big)\,. \tag{78}$$

It follows that

$$\mathrm{Tr}\Big(\hat{\rho}\,\mathbf{S}(\mathbf{r})\Big) = \frac{\displaystyle\sum_{n=1}^{3} \mathbf{S}_n\, \mathrm{e}^{-\beta\,\mathbf{S}_n\cdot\mathbf{B}(\mathbf{r},\tau)}}{1+\displaystyle\sum_{n=1}^{3} \mathrm{e}^{-\beta\,\mathbf{S}_n\cdot\mathbf{B}(\mathbf{r},\tau)}}\;, \tag{79}$$

is actually a self-consistency equation that we have to solve. In practice, we start from a randomly chosen configuration $\langle\,\mathbf{S}(\mathbf{r})\,\rangle$, which determines $B(\mathbf{r},\tau)$ of Supplementary Eq. (78) [11], calculate by means of (79) the new configuration $\langle\,\mathbf{S}(\mathbf{r})\,\rangle$, and proceed till convergence. Such mean-field scheme well inside the monoclinic insulator phase does not even require enforcing the curl-free condition $\boldsymbol{\nabla}\wedge\boldsymbol{\epsilon}(\mathbf{r})=0$. Indeed, because of the Z_3 symmetry among the three allowed monoclinic $\mathbf{S}_n$, $n=1,2,3$, the interface between two domains identified by two of those pseudo-spins turns out to be automatically directed along the third one.

Evidently, both the pseudo-spin representation and the chosen mean-field scheme are valid only inside the monoclinic phase well below the rhombohedral metal spinodal point. Here, in fact, the monoclinic minima of the energy functional are deep enough to justify neglecting amplitude fluctuations.

SUPPLEMENTARY FIGURES

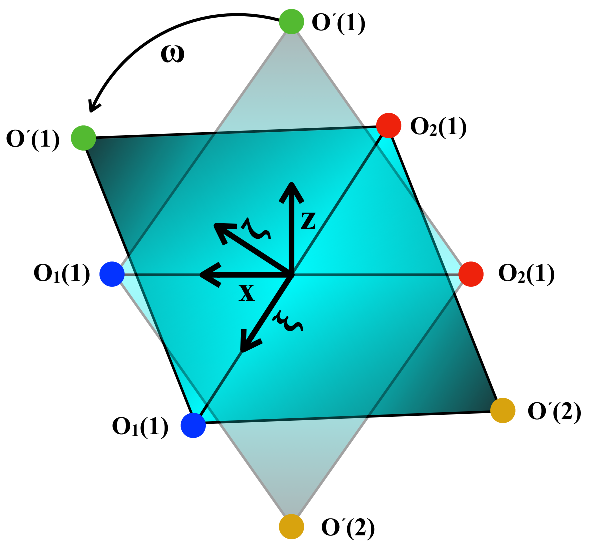

Supplementary Figure 1. Rotation around y of the octahedron.

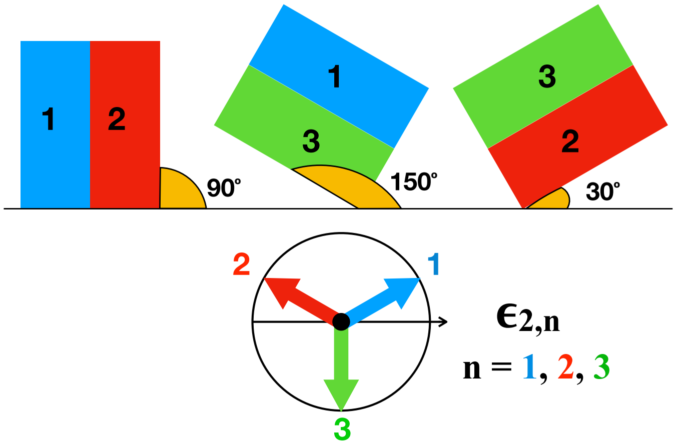

Supplementary Figure 2. Possible interfaces

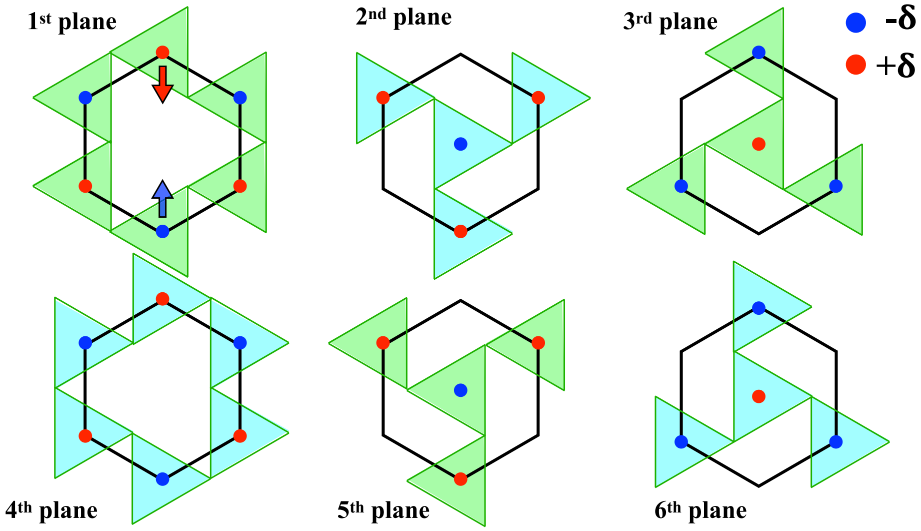

Supplementary Figure 3. Positions of V and O for each of the six layers in the hexagonal cell. V atoms shifted below the basal plane are denoted as blue circles, while those shifted up are in red. The vertices of the triangles are the positions of the oxygens on top of the corresponding plane. The arrows indicate the motion of V in the monoclinic phase, which is the same for all planes. Namely, all red circles move down, while all blue ones up.

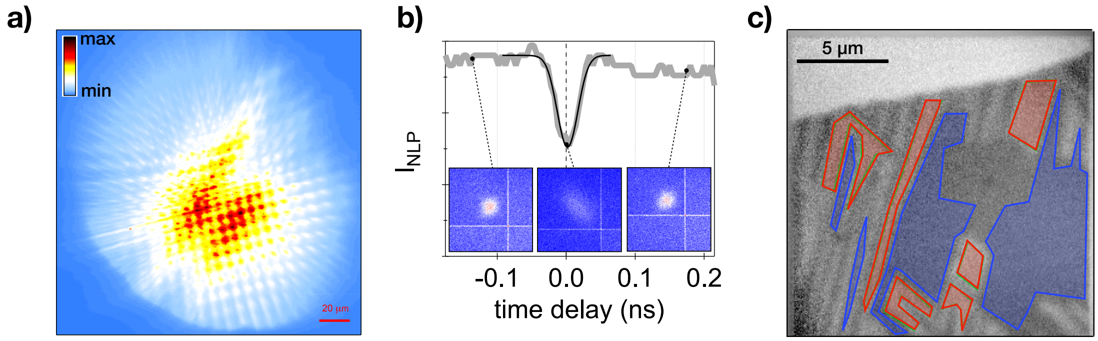

Supplementary Figure 4. **Temporal and spatial characterization of laser and X-rays beams for tr-PEEM experiment.** (a) Laser spot profile as reconstructed by means of non-linear photoemission map from a surface defect. (b) Intensity of the non-linear photoemission signal $I_{\rm NLP}$(t) from a surface defect as a function of the delay between the laser and X-ray fields. The minimum of the total intensity is shown in correspondence with the temporal coincidence between the near-IR pump and X-ray probe (i.e. time zero). Bottom panels: images of the integrated area used to obtain the total intensity $I_{\rm NLP}$(t). (c) Example of the tr-PEEM image obtained from the acquisition protocol. The red and blue areas represent, respectively, the regions of maximum (I^{light}) and minimum (I^{dark}) photoemission intensity contrast used to extract the relative contrast variation $\delta I(t)$ between neighbouring monoclinic domains.

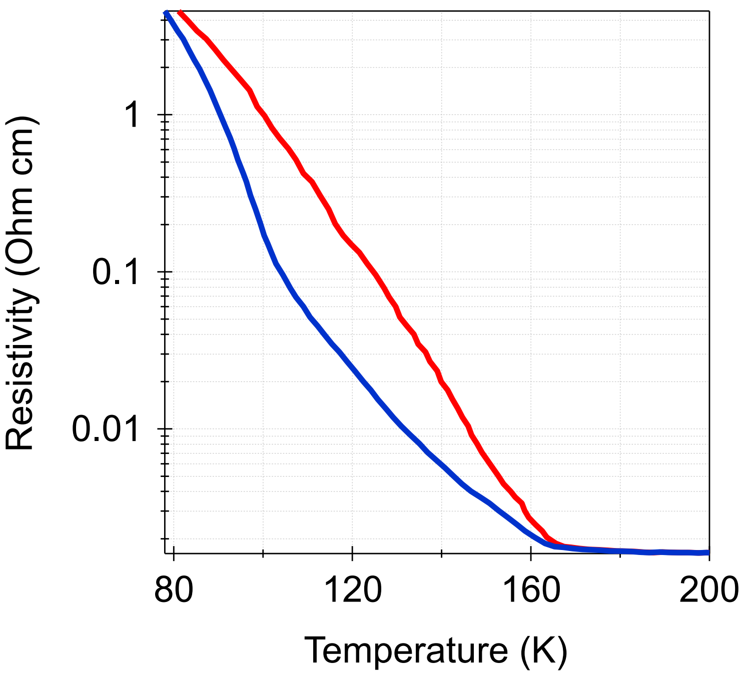

Supplementary Figure 5. Typical equilibrium resistivity hysteresis measured as function of the temperature of a 50 nm V_2O_3 on (0001)-Al_2O_3 substrate. The red (blue) curves indicate the heating (cooling) branch. The curves have been measured using the 4-points Van Der Pauw configuration with a temperature sweep rate of 0.5 K/min.

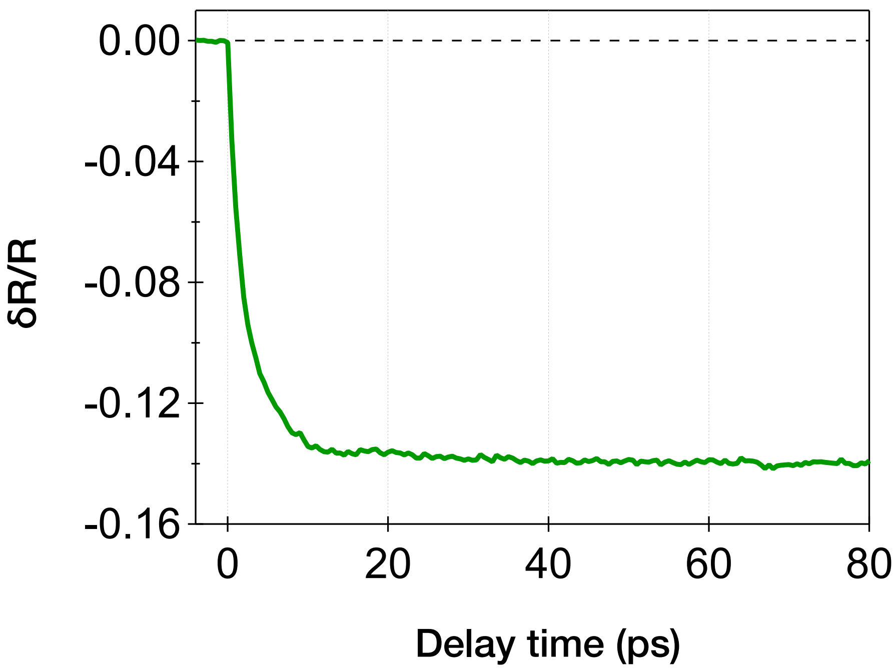

Supplementary Figure 6. Typical transient variation of reflectivity $\delta R/R$ measured at T=100 K as function of the time delay between pump and probe pulses. The pump (probe) wavelength is 1.5 eV (2.4 eV). The repetition rate is 10 kHz to avoid Joule heating effects. The incident pump fluence is 8 mJ/cm^2, enough to completely photo-induce the metallic state as shown in Fig. 8d of the main text.

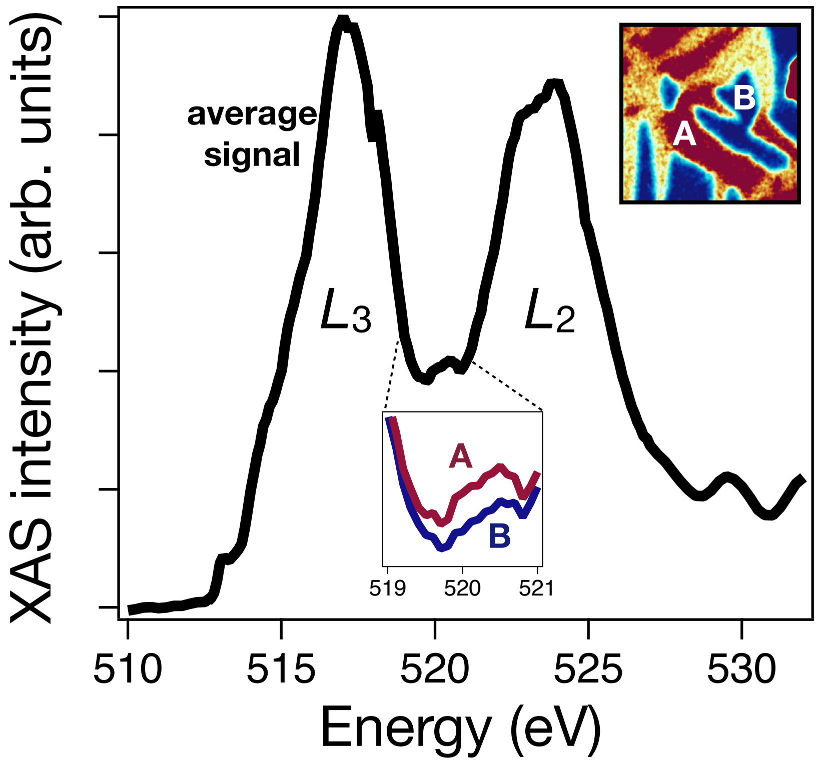

Supplementary Figure 7. **XAS contrast**. X-ray Absorption Spectroscopy (XAS) spectra showing the $L_{2,3}$ vanadium absorption lines. The black solid line is the average XAS signal obtained by averaging over the entire image. The bottom central inset shows the different XAS signal from regions (A, B) corresponding to different monoclinic domains, as indicated in the top right inset. The difference of the two signal corresponds to the XAS contrast between different spatial regions as captured by PEEM.

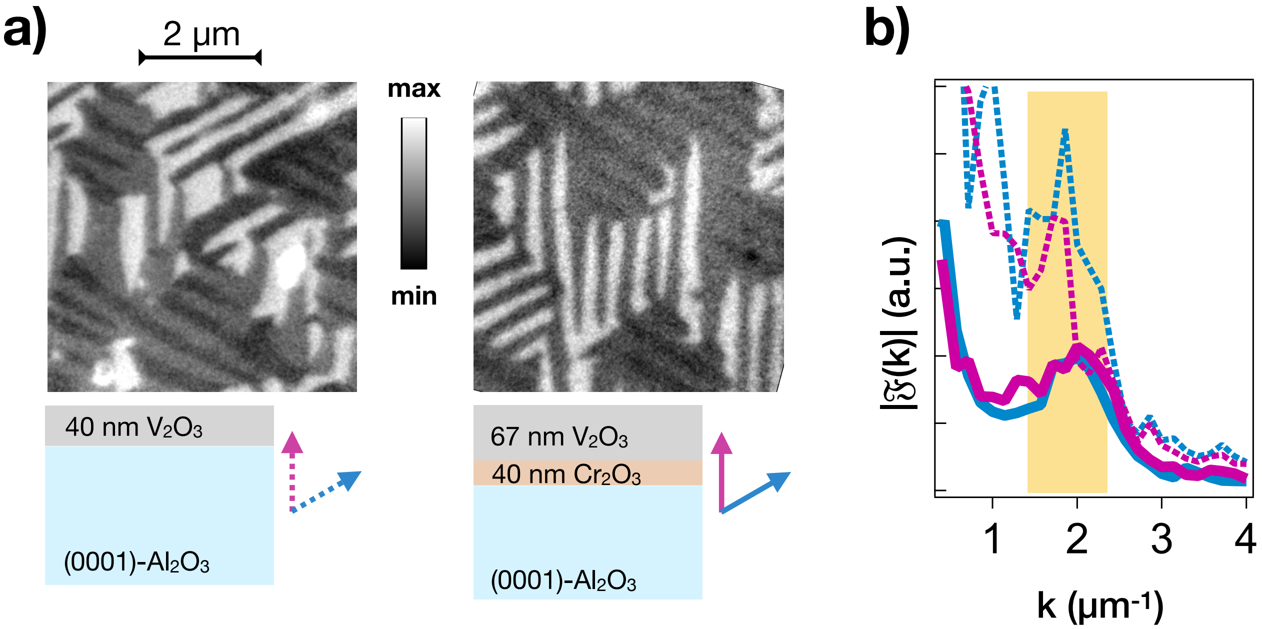

Supplementary Figure 8. **Domains morphology for different V_2O_3 samples**. a) PEEM images taken at 120 K for the sample with (right) and without (left) the Cr_2O_3 buffer layer. In the bottom we report schematics of the sample structure. The dashed/solid coloured arrows (blue, pink) highlight the main orientations of the monoclinic domains. b) Cuts of the two-dimensional Fast Fourier Transform along the directions defined by the arrows in panel a). The yellow box highlights the main peaks (in k-space) corresponding to the periodicity of the stripe domains in the real space.

SUPPLEMENTARY REFERENCES

[1] L. Dillemans, T. Smets, R. R. Lieten, M. Menghini, C.-Y. Su, and J.-P. Locquet, Applied Physics Letters **104**, 071902 (2014).

[2] M. G. Brik, K. Ogasawara, H. Ikeno, and I. Tanaka, The European Physical Journal B - Condensed Matter and Complex Systems **51**, 345 (2006).

[3] J. Wachtman, J B, W. E. Tefft, J. Lam, D G, and R. P. Stinchfield, Journal of research of the National Bureau of Standards. Section A, Physics and chemistry **64A**, 213 (1960).

[4] D. N. Nichols, R. J. Sladek, and H. R. Harrison, Phys. Rev. B **24**, 3025 (1981).

[5] H. Yang, R. Sladek, and H. Harrison, Solid State Communications **47**, 955 (1983).

[6] H. Yang and R. J. Sladek, Phys. Rev. B **34**, 2627 (1986).

[7] S. R. Shenoy, T. Lookman, A. Saxena, and A. R. Bishop, Phys. Rev. B **60**, R12537 (1999).

[8] P. Pfalzer, G. Obermeier, M. Klemm, S. Horn, and M. L. denBoer, Phys. Rev. B **73**, 144106 (2006).

[9] S. R. Shenoy and T. Lookman, Phys. Rev. B **78**, 144103 (2008).

[10] R. Vasseur, T. Lookman, and S. R. Shenoy, Phys. Rev. B **82**, 094118 (2010).

[11] In reality, we find more convenient to calculate $\langle \boldsymbol{S}(\mathbf{q}) \rangle$ from $\langle \mathbf{S}(\mathbf{r}) \rangle$ by fast Fourier transform (FFT), and then calculate $B(\mathbf{r})$ of Eq. (78) by inverse FFT.
